# Supplementary material for: P-TRAP: a Panicle Trait Phenotyping tool
Source: BMC Plant Biol. 2013 Aug 29;13:122. doi: 10.1186/1471-2229-13-122 (PMC3848748; doi:10.1186/1471-2229-13-122)
Supplement: Additional file 7 — P-TRAP source code. The Netbeans project that contains the source code of the application. [file 1471-2229-13-122-S7.zip › javadoc.zip!/javadoc/org-basmati-workspace/org/basmati/workspace/Drawer.html]

Drawer (WorkSpace)


JavaScript is disabled on your browser.


- Package
- Class
- Tree
- Deprecated
- Index
- Help

*org.basmati.workspace 1.0*

- Prev Class
- Next Class

- Frames
- No Frames

- All Classes

- Summary:
- Nested |
- Field |
- Constr |
- Method

- Detail:
- Field |
- Constr |
- Method


org.basmati.workspace

## Class Drawer

- java.lang.Object
- - org.basmati.workspace.Drawer

- ---

    

  ```
  public abstract class Drawer
  extends java.lang.Object
  ```

  Superimpose graph and particles on a given image

  Author:
  :   faroq

- - ### Constructor Summary

    Constructors

    Constructor and Description

    `Drawer()`
  - ### Method Summary

    Methods

    | Modifier and Type | Method and Description |
    | --- | --- |
    | `static java.awt.image.BufferedImage` | `deepCopy(java.awt.image.BufferedImage bi)` Create a copy of a given buffered image |
    | `static void` | `drawGraph(java.awt.Image image, org.bioalg.graphprocessor.GraphModel gm)` Super impose a graph on an image |
    | `static java.awt.image.BufferedImage` | `drawParticlesModel(java.awt.Image image, org.biolag.particleprocessor.ParticlesModel pm, java.awt.Color mainColor, boolean drawAnchors)` Draw a set of particles on a given image, the blue color is used. |

    - ### Methods inherited from class java.lang.Object

      `clone, equals, finalize, getClass, hashCode, notify, notifyAll, toString, wait, wait, wait`

- - ### Constructor Detail


    - #### Drawer

      ```
      public Drawer()
      ```
  - ### Method Detail


    - #### drawGraph

      ```
      public static void drawGraph(java.awt.Image image,
                   org.bioalg.graphprocessor.GraphModel gm)
      ```

      Super impose a graph on an image

      Parameters:
      :   `image` -
      :   `gm` -


    - #### drawParticlesModel

      ```
      public static java.awt.image.BufferedImage drawParticlesModel(java.awt.Image image,
                                                    org.biolag.particleprocessor.ParticlesModel pm,
                                                    java.awt.Color mainColor,
                                                    boolean drawAnchors)
      ```

      Draw a set of particles on a given image, the blue color is used.

      Parameters:
      :   `image` -
      :   `pm` -
      :   `mainColor` - the main color used for painting
      :   `drawAnchors` - if ture it draws the anchors of the particle (the four
          points of the crossing lines).


    - #### deepCopy

      ```
      public static java.awt.image.BufferedImage deepCopy(java.awt.image.BufferedImage bi)
      ```

      Create a copy of a given buffered image

      Parameters:
      :   `bi` - the input image.

      Returns:
      :   a clone of the input image.


- Package
- Class
- Tree
- Deprecated
- Index
- Help

*org.basmati.workspace 1.0*

- Prev Class
- Next Class

- Frames
- No Frames

- All Classes

- Summary:
- Nested |
- Field |
- Constr |
- Method

- Detail:
- Field |
- Constr |
- Method
